# Dermatitis and Aging-Related Barrier Dysfunction in Transgenic Mice Overexpressing an Epidermal-Targeted Claudin 6 Tail Deletion Mutant

**DOI:** 10.1371/journal.pone.0007814

**Published:** 2009-11-13

**Authors:** Tammy-Claire Troy, Azadeh Arabzadeh, Nathalie M. K. Larivière, Adebola Enikanolaiye, Kursad Turksen

**Affiliations:** 1 Sprott Centre for Stem Cell Research at the Ottawa Hospital Research Institute, Ottawa, Ontario, Canada; 2 Department of Cellular and Molecular Medicine, University of Ottawa, Ottawa, Ontario, Canada; 3 Divisions of Dermatology and Endocrinology, Department of Medicine, University of Ottawa, Ottawa, Ontario, Canada; 4 Division of Reproductive Endocrinology, Department of Obstetrics, Gynaecology, Faculty of Medicine, University of Ottawa, Ottawa, Ontario, Canada; Tufts University, United States of America

## Abstract

The barrier function of the skin protects the mammalian body against infection, dehydration, UV irradiation and temperature fluctuation. Barrier function is reduced with the skin's intrinsic aging process, however the molecular mechanisms involved are unknown. We previously demonstrated that Claudin (Cldn)-containing tight junctions (TJs) are essential in the development of the epidermis and that transgenic mice overexpressing Cldn6 in the suprabasal layers of the epidermis undergo a perturbed terminal differentiation program characterized in part by reduced barrier function. To dissect further the mechanisms by which Cldn6 acts during epithelial differentiation, we overexpressed a Cldn6 cytoplasmic tail deletion mutant in the suprabasal compartment of the transgenic mouse epidermis. Although there were no gross phenotypic abnormalities at birth, subtle epidermal anomalies were present that disappeared by one month of age, indicative of a robust injury response. However, with aging, epidermal changes with eventual chronic dermatitis appeared with a concomitant barrier dysfunction manifested in increased trans-epidermal water loss. Immunohistochemical analysis revealed aberrant suprabasal Cldn localization with marked down-regulation of Cldn1. Both the proliferative and terminal differentiation compartments were perturbed as evidenced by mislocalization of multiple epidermal markers. These results suggest that the normally robust injury response mechanism of the epidermis is lost in the aging Involucrin-Cldn6-CΔ196 transgenic epidermis, and provide a model for evaluation of aging-related skin changes.

## Introduction

Formed during development by a series of cell commitment, mesenchymal-epithelial cell interactions, and terminal differentiation, the mammalian epidermis undergoes continuous self-renewal in a tightly regulated process of epidermal cell proliferation and differentiation [1]–[3]. As the end result of terminal differentiation, the robust barrier function of the skin protects against microorganism invasion and UV irradiation, inhibits water loss, regulates body temperature and is an important part of the host defense system [4]. These important functions decline in efficiency with aging, leading to an inefficient epidermal injury response and dermatitis [5]–[7], for reasons that are not yet understood.

Tight junctions (TJs) are essential not only for dividing epidermal cells into apical and basolateral compartments to create cell polarity [8], but also for the existence of skin barrier function by regulating the selective permeability of the paracellular pathway [9]–[11]. The selectivity function of TJs is imparted by Claudins (Cldns), a family of 23 highly conserved tetraspan membrane proteins whose heterogeneity stems in large part from distinctly charged amino acid sequences in the first external loop [11]–[13]. Cldn type and mixing ratio thus provide for the specific permeability requirements of different epithelia [12]. The importance of Cldns in epidermal differentiation and barrier function has been confirmed by experiments in which Cldn expression has been perturbed in epidermal cells; for example, Cldn1 knockout mice die shortly after birth due to skin barrier dysfunction [14]. Involucrin-Cldn6 (Inv-Cldn6) transgenic mice also suffer skin barrier dysfunction, the severity/lethality of which is dependent upon the level of Cldn6 overexpression [15], [16]. Further, Inv-Cldn6-CΔ187 transgenic mice overexpressing a cytoplasmic tail-ablated Cldn6 display epidermal hyperproliferation, apparently due to an inefficiency of Cldn protein membrane targeting, as a result of the unfolded protein response pathway [17].

The latter data suggest the importance of the cytoplasmic tail portion of Cldn molecules in cell signaling during epidermal differentiation. The cytoplasmic tail of different Cldns, while relatively constant in length, is divergent in sequence, but a number of putative functional protein domains are present in many family members [12], [18]. To address the activities of the functional domains in more detail, we again used the involucrin promoter (Inv) this time to target a shorter deletion in the cytoplasmic tail (Cldn6-CΔ196) to the differentiative compartment of the epidermis. The Inv-Cldn6-CΔ196 transgenic mice possess subtle epidermal differentiation abnormalities at birth that by 1-month of age are completely normalized. However, with aging, Inv-Cldn6-CΔ196 mice suffered dermatitis, often manifested as patent wounds in repetitive grooming areas. Normal hydration levels were not maintained in the aging skin, and immunohistochemistry revealed perturbations in the expression and localization of multiple Cldns, as well as various classical markers of epidermal differentiation. These results suggest that the normally robust injury response mechanism of the epidermis is lost in the aging Inv-Cldn6-CΔ196 transgenic epidermis and provides a model for evaluation of chronic dermatitis and aging-related skin changes.

## Methods

### Generation of Inv-Cldn6-CΔ196 transgenic mice

The Inv-Cldn6-CΔ196 construct [19] was injected into ova collected from superovulated CD1 mice to generate viable lines at the Ottawa Hospital Research Institute (OHRI) Transgenic Mouse Facility as previously described [20]. Genotyping was performed by PCR using genomic DNA and primers specific for Cldn6 (5′-ATG GCC TCT ACT GGT CTG CA-3′) and the FLAG® tag (5′-TCA CTT GTC ATC GTC GTC CTT G-3′). Age-matched wild type and Inv-Cldn6-CΔ196 transgenic mice were photographed using a Nikon COOL-PIX950 digital camera (Nikon) for image processing with Adobe Photoshop version 7.0 (Adobe Systems). All research complied with the principles and guidelines of the Canadian Council on Animal Care and was approved by the Ottawa Hospital Research Institute Animal Care Committee. Once the dermatitis phenotype became unmanageable, Inv-Cldn6-CΔ196 transgenic mice were euthanized according to institutional and legislative policies.

### Sample preparation, histology and immunohistochemistry

#### a) Sample collection

Freshly dissected skin samples (∼1 cm^2^) were collected at various postnatal time points (birth, 1 week, 2 weeks, 3 weeks, 1 month, 2 months, 3 months, 5 months, 6 months) from either the mid-dorsal region or areas of frequent grooming (e.g. the neck and behind the ears) of Inv-Cldn6-CΔ196 transgenic mice and their age-matched CD1 counterparts.

#### b) Frozen sections

Frozen sections were required for immunolocalization with FLAG® antibodies, whereas histology and all other immunostaining procedures were performed using paraffin-embedded sections. For frozen sections, skin samples were embedded in HistoPrep (Fisher Scientific), solidified using dry ice-chilled isopentane and 5 µm sections were mounted onto Superfrost®/Plus slides (Fisher Scientific) as described [17]. Samples were fixed for 10 minutes in methanol (−20°C) and washed with PBS before immunohistochemistry (see below).

#### c) Paraffin sections and histology

For paraffin sections, skin samples were fixed overnight in Bouin's solution (75% saturated picric acid, 20% formaldehyde, 5% glacial acetic acid) and dehydrated via a graded series of ethanol washes (from 30% to 100%) before paraffin embedding and sectioning (5 µm). Sections mounted onto Superfrost®/Plus slides (Fisher Scientific) were dewaxed using toluene and rehydrated in a reverse graded series of ethanol washes to water. Following antigen unmasking and washing in PBS, sections were either stained with hematoxylin and eosin (H&E) as described [15] or further processed for immunohistochemistry (see below).

#### d) Immunohistochemistry

After blocking for non-specific antibody binding (10% goat serum, 0.8% BSA, 1% gelatin in PBS), skin samples were incubated for 1 hour in antibodies appropriately diluted in incubation buffer (1% goat serum, 0.8% BSA, 1% gelatin in PBS) [15]. Antibodies recognizing the following proteins were used: FLAG^®^ (M2 monoclonal) (1∶440; Sigma), K15 (1∶100; rabbit #UC55), K5 (1∶100; rabbit #5054), K14 (1∶100; rabbit #199), K1 (1∶100; rabbit #UC81), K6 (1∶200; BabCO), K17 (1∶500; a gift from Dr. Pierre Coulombe, Johns Hopkins), involucrin (1∶100; BabCO), filaggrin (1∶100; BabCO), loricrin (1∶100; rabbit #UC84), Cldn1 (6∶100; Zymed Laboratories), Cldn2 (1∶200; Zymed Laboratories), Cldn3 (1∶50; Zymed Laboratories), Cldn5 (1∶100; Zymed Laboratories), Cldn6 (1∶100; hen #3677), Cldn11 (1∶100; hen #3680), Cldn12 (1∶100; hen #5186) and Cldn18 (1∶100; rabbit #A9953). Following washes (0.8% BSA, 1% gelatin in PBS), skin samples were incubated for 1 hour at room temperature with FITC-conjugated secondary antibodies against mouse, rabbit and chicken (1∶50; Jackson ImmunoResearch) diluted in incubation buffer. After washes in wash buffer and PBS, sections were mounted with Moviol 4–88 (Calbiochem) containing 2.5% 1,4 diazobicyclo-[2,2,2]-octane (DABCO; Sigma) for observation on a Zeiss Axioplan 2 fluorescence microscope equipped with an AxioCam camera and Axio Vision 2.05 software (Carl Zeiss); digital images were processed using Adobe Photoshop version 7.0 (Adobe Systems).

### Protein isolation and immunoblotting

Skin samples (0.4 g) were dissected from either the mid-dorsal region or areas of frequent grooming (e.g. the neck and behind the ears) of Inv-Cldn6-CΔ196 transgenic mice and their age-matched CD1 counterparts and whole cell protein extracts were prepared by homogenizing in SDS extraction buffer (62.5 mM Tris pH 6.8, 25% glycerol, 2% SDS and 2% β-mercaptoethanol with pepstatin A and a complete mini protease inhibitor cocktail tablet [Roche]) followed by centrifugation. Following a 30-minute incubation in sample reducing buffer (62.5 mM Tris pH 6.8, 25% glycerol, 2% SDS, 0.1% bromophenol blue and 2% β-mercaptoethanol), boiling and centrifugation, 10 µg of soluble proteins were separated on 12% SDS-polyacrylamide gels and transferred to nitrocellulose [17]. After blocking for non-specific antibody binding (5% skim milk, TBS/0.1% tween-20 [TBS-T]), blots were incubated at 4°C overnight with diluted (5% skim milk, TBS-T) antibodies against FLAG® (polyclonal) (1∶500; Sigma) and GAPDH (1∶20,000; Abcam). After several washes in TBS-T, blots were incubated in diluted (5% skim milk, TBS-T) HRP-conjugated antibodies against mouse and rabbit IgG (1∶20,000; Amersham) for 1 hour at room temperature and washed with TBS-T before incubation with chemiluminescent HRP substrate (Millipore) and visualization on BioMax XAR autoradiography film (Kodak). Films were scanned and digital images were prepared using Adobe Photoshop version 7.0 (Adobe Systems).

### Barrier integrity assay

Trans-epidermal water loss (e.g. impedence) of both Inv-Cldn6-CΔ196 transgenic and wild type mouse skin was measured using a dermal phase meter (DPM) (Nova Technology Corporation) as described [16]. Diminished barrier integrity is reflected in higher DPM values over time as depicted digitally with EDWINA software (Nova Technology Corporation) and converted into graphical form with Excel software (Microsoft).

### Cornified envelope extracts

Using dorsal skin samples of CD1 and Inv-Cldn6-CΔ196 transgenic mice, purified cornified envelope extracts were prepared as described [16], [21], [22]. Briefly, skin samples were immersed in hot extraction buffer (0.1 M Tris-HCl pH 8.5, 2% SDS, 20 mM dithiothreitol, 5 mM EDTA), followed by 15-minute incubation at 95°C and gentle centrifugation before observation using an Olympus CK2 inverted microscope (Olympus) equipped with a Nikon COOLPIX 4500 digital camera (Nikon). Images were produced with Adobe Photoshop version 7.0 (Adobe Systems).

## Results

### Generation and expression analysis of Inv-Cldn6-CΔ196 transgenic mice

**Figure 1:**
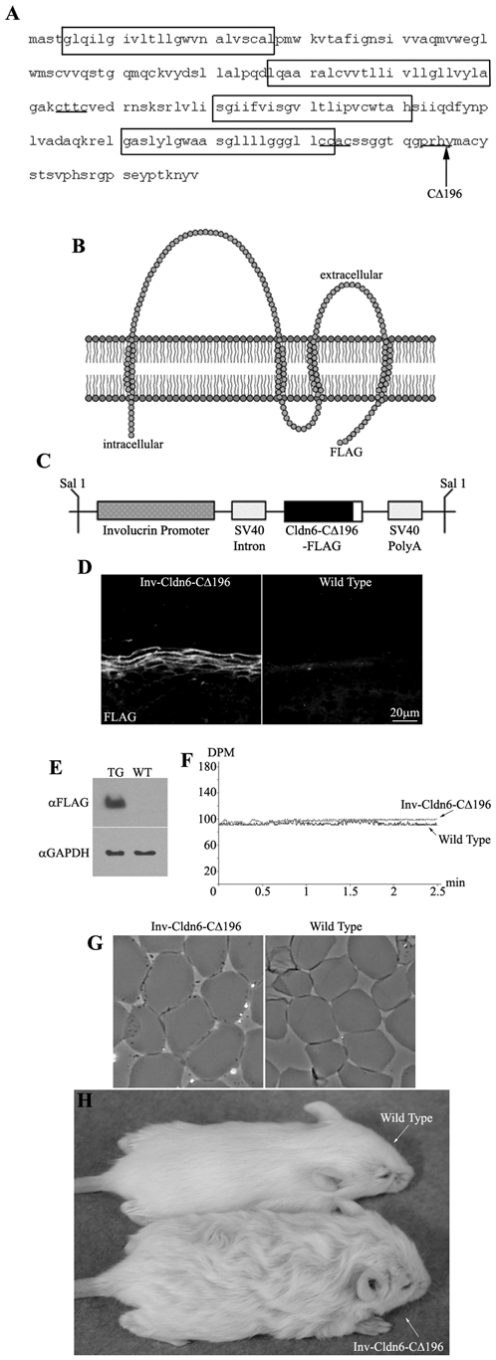
Inv-Cldn6-CΔ196 transgenic mice. The Cldn6 protein sequence is shown; transmembrane-spanning regions are encased within boxes, the CXXC motifs are underlined, and the truncation site is indicated with an arrow (**A**). The Inv-Cldn6-CΔ196 mutant was created by deleting the C-terminal half of the cytoplasmic tail domain of Cldn6 after amino acid 196 (**B**). The Inv promoter was use to drive transgene expression to the suprabasal compartment of the transgenic mouse epidermis, where TJs are localized (**C**). Transgene expression was confirmed to be restricted to the upper spinous and granular layers of the epidermis as visualized by immunohistochemistry using anti-FLAG antibodies (**D**) and immunoblotting confirmed a ∼19.5kDa band corresponding to the transgene product in skin samples from transgenic (TG) but not wild type (WT) skin samples using anti-GAPDH as a loading control (**E**). Trans-epidermal water loss measurements confirmed no skin barrier dysfunction in the Inv-Cldn6-CΔ196 transgenic mice at birth (**F**). This was further supported by evaluation of cornified envelopes; which were characterized by a rigid shape and uniform size comparable to that of the wild type (**G**). Inv-Cldn6-CΔ196 mice were easily identifiable by their frizzed and lackluster coat appearance as compared to the wild type, a phenotype that persisted throughout life (**H**).

We previously reported that overexpression of a Cldn6 lacking the C-terminal half of the cytoplasmic tail domain (e.g. truncation after amino acid 196; Cldn6-CΔ196) (**Figure 1A-C**) in the suprabasal compartment of the mouse epidermis results in marked hyperproliferative squamous invaginations/cysts replacing hair follicles and lethal epidermal barrier dysfunction [19]. The rationale for generating the Δ196 mutation was based on two main factors. First, we already knew that tailless Cldn6 was not targeting to the membrane appropriately. Second, we wanted to delete the PDZ domain that is at the tip of the cytoplasmic tail as well as putative phosphorylation sites, which could be accomplished by deletion from amino acid 196. The choice of 196 rather than 195 or 197, both of which would also have accomplished removal of the PDZ and putative phosphorylation sites, was one of convenience for the steps required to make our mutant insert. Because we have demonstrated previously that the level of Cldn6 overexpression correlates to the severity of the phenotype [15], [16], we re-derived F0s with lower Cldn6-CΔ196 expression to avoid lethality and explore the function of the Cldn6 tail in postnatal animals; levels of expression of the mutant Cldn6 is half of that observed in our previously generated mice. Nine Inv-Cldn6-CΔ196 positive F0 transgenic mice (five females and four males) were generated and three viable lines exhibiting indistinguishable phenotypes were successfully established. Cldn6-CΔ196 expression was confirmed to be confined to the upper spinous and granular layers of the epidermis and localized to cell membranes, mimicking endogenous Cldn6 expression (**Figure 1D**). Immunoblotting confirmed a ∼19.5kDa band corresponding to the transgene product in skin samples from Inv-Cldn6-CΔ196 but not wild type littermates (**Figure 1E**).

Inv-Cldn6-CΔ196 neonates displayed no gross phenotypic anomalies compared to their wild type counterparts; trans-epidermal water loss (TEWL) measurements (DPM in the range of 98–105) (**Figure 1F**) and the absence of X-Gal (5-bromo-4-chloro-3-indolyl-β-D-galactopyranoside) penetration (not shown) confirmed no skin barrier dysfunction in the transgenic mice at birth. Extracts from Inv-Cldn6-CΔ196 mouse skin revealed normal looking cornified envelopes, with a rigid shape and uniform size (**Figure 1G**), providing further evidence that early postnatal barrier function was not perturbed. As was observed in our Inv-Cldn6 and Inv-Cldn6-CΔ187 mouse models [15], [23], however, the emergence of hair fibers provided a diagnostic identifier for Inv-Cldn6-CΔ196 transgenic mice. In comparison to the sleek and lustrous appearance of wild type mice, the coat of Inv-Cldn6-CΔ196 mice was frizzed and lackluster, a phenotype that persisted throughout life (**Figure 1H**); however since it does not appear to have any direct relevance to the aging-related skin barrier defects reported herein, it has not been further explored.

### Epidermal maturation is delayed in Inv-Cldn6-CΔ196 mice

**Figure 2:**
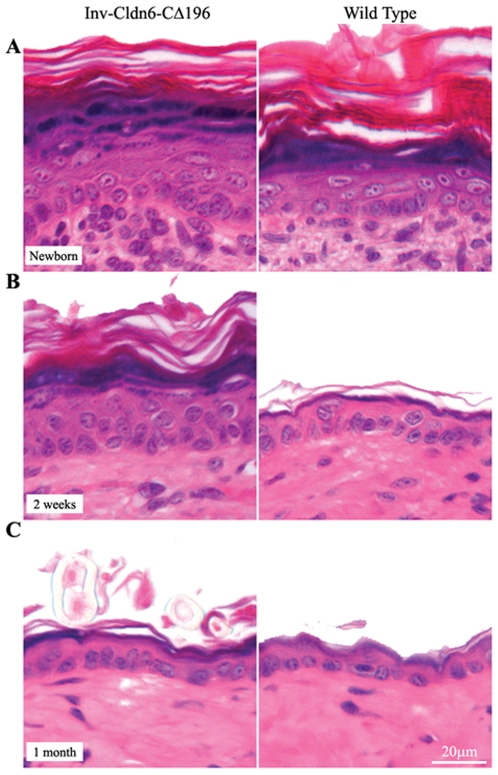
Histological abnormalities in the Inv-Cldn6-CΔ196 transgenic epidermis. Histological analysis of newborn (**A**) Inv-Cldn6-CΔ196 transgenic mice as compared to the wild type revealed a moderately thicker epidermis in transgenic samples, with a thicker stratum corneum, a less-compacted granular layer and an overall expanded suprabasal compartment. At 2-weeks of age (**B**), the Inv-Cldn6-CΔ196 epidermis remained somewhat less mature and thicker than that of the wild type; however by 1-month of age (**C**) the transgenic and wild type samples were histologically indistinguishable.

Although in the epidermis there were no gross phenotypic abnormalities or barrier dysfunction apparent at birth, histological examination revealed that the newborn (**Figure 2A**) and 1 week-old (not shown) transgenic epidermis was moderately thicker than that of the wild type, with a thicker stratum corneum, a less-compacted granular layer and an overall expanded suprabasal compartment. By 2-weeks of age, the Inv-Cldn6-CΔ196 epidermis displayed a more compact and organized granular layer, but it remained less mature and thicker than that in the wild type animals (**Figure 2B**). Typical epidermal thinning had commenced in the transgenic epidermis by 3-weeks of age (∼1 week later than wild type; not shown) and by 1-month of age the Inv-Cldn6-CΔ196 transgenic epidermis was histologically indistinguishable from that of the wild type (**Figure 2C**).

**Figure 3:**
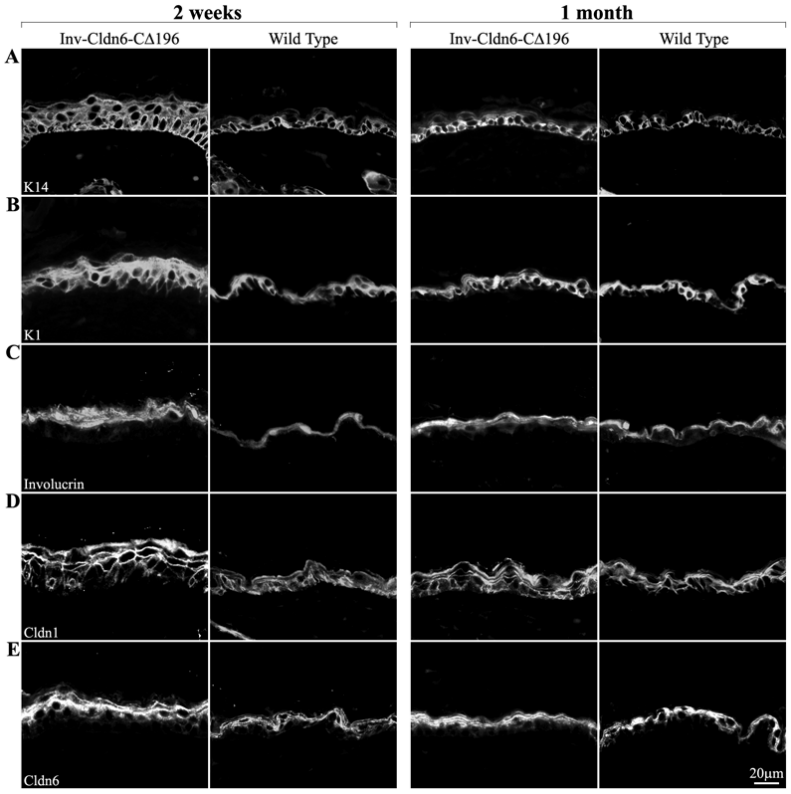
Perturbation of markers of epidermal differentiation. Immunofluorescence was used to evaluate various markers of epidermal differentiation including K14 (**A**), K1 (**B**), involucrin (**C**), Cldn1 (**D**) and Cldn6 (**E**) in Inv-Cldn6-CΔ196 mice as compared to their age-matched wild type counterparts. In each case abnormalities were present in samples from 2-week old (left panel) transgenic mice that were normalized by 1-month of age (right panel). For example, the K14 expression zone was extended into the suprabasal compartment the Inv-Cldn6-CΔ196 epidermis until epidermal thinning was achieved. In addition, broadened K1 and involucrin expression compartments was seen early but normalized by 1-month of age. Sporadic Cldn1-positive basal cells were evident in the 2-week old transgenic epidermis; by 1-month of age Cldn1-positive cells occupied both the basal and suprabasal compartments reminiscent of the wild type. Cldn6, a suprabasal-specific Cldn, was observed in an expanded zone early on, and was normalized by 1-month of age.

Consistent with the histological results, abnormalities in the expression of epidermal differentiation markers were present in neonatal Inv-Cldn6-CΔ196 mice but these also normalized by 1-month of age (**Figure 3**; the transgenic and wild type epidermis after 2 weeks and 1 month are shown). For example, K5 and K15 were restricted to basal cells at all time points in both the wild type and Inv-Cldn6-CΔ196 epidermis (not shown), but K14, another basal compartment marker, occupied an expanded zone extending into the suprabasal compartment at birth and until epidermal thinning was achieved in the Inv-Cldn6-CΔ196 epidermis (**Figure 3A**). The suprabasal layers of the epidermis normally express K1 [24], and when in a state of hyperproliferation, K6 and K17 [25]–[27]. While neither K6 nor K17 was expressed throughout the time analyzed (not shown), a broadened compartment of K1-positive cells, consistent with the increased number of suprabasal cell layers observed histologically, was seen early but normalized by 1-month of age in the Inv-Cldn6-CΔ196 transgenic epidermis (**Figure 3B**). Similarly the expression compartments of various structural proteins in the stratum corneum, including involucrin (**Figure 3C**), filaggrin and loricrin (not shown), were also expanded in the juvenile Inv-Cldn6-CΔ196 epidermis, and a tightly compacted stratum corneum was not observed in the transgenic epidermis until thinning comparable to that in the wild type was achieved.

In parallel with changes in epidermal markers, the thickened newborn and 1-week-old Inv-Cldn6-CΔ196 epidermal basal layer was devoid of Cldn1, which was clearly present in the wild type epidermis (not shown). However, after 2 weeks, sporadic Cldn1-positive basal cells were evident, and the frequency increased as thinning progressed until 1-month of age when Cldn1-positive cells completely occupied the basal and suprabasal compartments in both the transgenic and wild type mice (**Figure 3D**). The suprabasal-specific Cldns, Cldn6 (**Figure 3E**), Cldn11, Cldn12 and Cldn18 (not shown) were seen in the expanded suprabasal region of the Inv-Cldn6-CΔ196 epidermis; each Cldn evaluated maintained a strictly membranous localization and normalized by 1-month to a zone comparable to wild type. Cldns that are not normally expressed in the epidermis (e.g. Cldn2, Cldn3 and Cldn5) [16] were not observed in either the wild type or Inv-Cldn6-CΔ196 epidermis at any time evaluated (not shown). The data suggest that Inv-Cldn6-CΔ196 transgenic mice are born with differentiation abnormalities leading to slower epidermal maturation, but these are too mild to manifest in skin barrier defects or death, and are repaired between birth and 1-month of age.

### Inv-Cldn6-CΔ196 mice experience age-related perturbations in epidermal differentiation and injury repair resulting in chronic dermatitis

**Figure 4:**
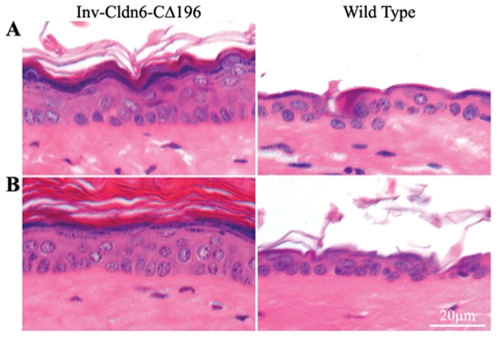
Histological evidence of epidermal abnormalities in the aging Inv-Cldn6-CΔ196 transgenic epidermis. Histological evidence of epidermal abnormalities emerged in aging Inv-Cldn6-CΔ196 transgenic mice after 3 (**A**) and 6 (**B**) months as compared to the wild type. These included an expanded stratum corneum, as well as an expanded suprabasal zone and a less compacted granular layer suggesting an intrinsic propensity for injury and inefficient repair that increases with aging in the Inv-Cldn6-CΔ196 epidermis.

Although by 1-month of age the Inv-Cldn6-CΔ196 epidermis was morphologically and biochemically indistinguishable from the wild type (**Figure 2** **and** **Figure 3**), as early as 2-months (not shown) of age, the transgenic epidermis showed histological evidence of thickening that persisted, and was somewhat exacerbated, at 3 (**Figure 4A**) and 6 (**Figure 4B**) months. At the latter ages, an expanded stratum corneum, increased number of suprabasal/spinous cell layers and a less compacted granular layer were clearly present, and reminiscent of the 2-week-old Inv-Cldn6-CΔ196 epidermis (see above).

**Figure 5:**
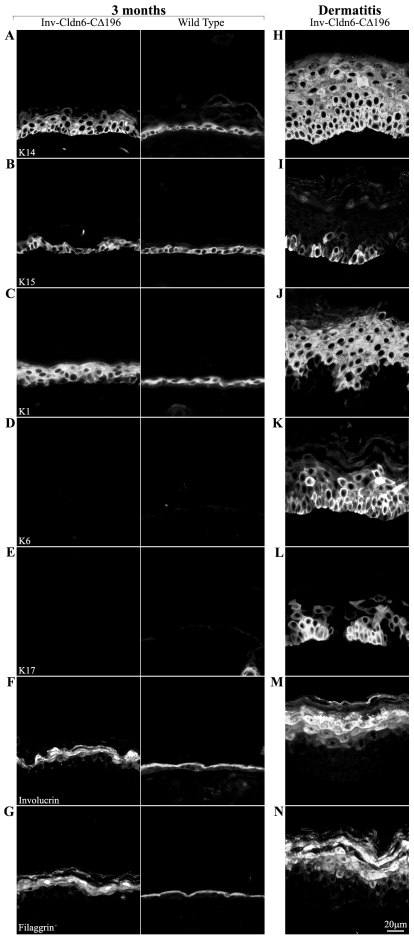
Aberrance in markers of epidermal differentiation during aging. Changes in the epidermal differentiation program of the aging Inv-Cldn6-CΔ196 transgenic epidermis were evaluated by immunofluorescence and compared to their age-matched wild type counterparts (samples from 3-month-old mice are shown – left panel); their expression/localization in dermatitis-affected areas is also indicated (right panel). K14 was expressed throughout the basal and suprabasal compartments in the aging Inv-Cldn6-CΔ196 epidermis (**A**), while K15 remained restricted to basal cells (**B**). The zone of K1 expression was expanded in the 3-month-old transgenic epidermis (**C**); however, neither K6- (**D**) nor K17-positive (**E**) cells were observed. An expanded zone of expression, and improper packing, of involucrin (**F**) and filaggrin (**G**) suggested perturbations in terminal differentiation program of the aged Inv-Cldn6-CΔ196 epidermis. In the dermatitis-affected areas, K14 (**H**) expression was expanded far into the suprabasal compartment, while K15 was only sporadically observed (**I**). A punctate K1 localization was evident throughout the thickened suprabasal zone of the dermatitis-affected Inv-Cldn6-CΔ196 epidermis (**J**), and K6- (**K**) and K17-positive (**L**) basal and suprabasal cells were observed. In addition, involucrin (**M**) and filaggrin (**N**) expression compartments were also expanded, with obvious packing defects evident.

The histological results suggested that intrinsic changes occurred in the transgenic epidermis, a possibility supported by the perturbed expression of differentiation markers; because results were similar from 2- to 6-months of age, we report results from 3-month-old Inv-Cldn6-CΔ196 transgenic mice only (**Figure 5**). K14 was expressed throughout the basal and suprabasal compartments in the aging Inv-Cldn6-CΔ196 epidermis (**Figure 5A**), while K5 (not shown) and K15 (**Figure 5B**) remained restricted to basal cells as in the wild type epidermis. K1 (**Figure 5C**) was seen throughout an expanded suprabasal zone in the transgenic epidermis corresponding to the increased number of spinous cell layers; however, neither K6- (**Figure 5D**) nor K17-positive (**Figure 5E**) interfollicular epidermal cells were observed nor was there any up-regulation in the expression of Ki67 or CD3 (not shown), suggesting no detectable hyperproliferation or immune cell infiltration. The localization of involucrin (**Figure 5F**), loricrin (not shown) and filaggrin (**Figure 5G**) suggested that there were also perturbations in terminal differentiation in the aged Inv-Cldn6-CΔ196 epidermis, with each marker occupying an expanded zone of expression consistent with the abnormal morphological appearance of the stratum corneum described above.

**Figure 6:**
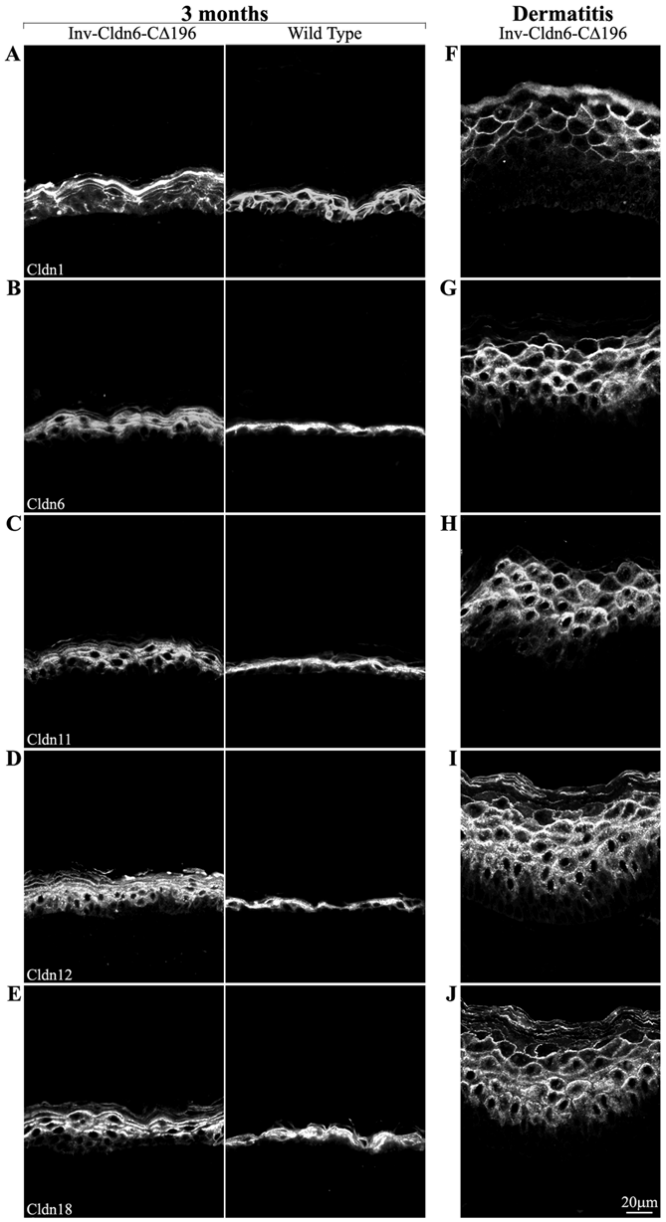
Evaluation of Cldns in the aging Inv-Cldn6-CΔ196 transgenic epidermis. The expression/localization of Cldns was also perturbed in the aging Inv-Cldn6-CΔ196 transgenic epidermis (samples are from 3-month-old mice – left panel); the dermatitis-affected zones are also shown (right panel). Cldn1-positive basal and suprabasal cells were reduced in the aging Inv-Cldn6-CΔ196 epidermis (**A**). In addition, a shift away from a strictly membrane localization and an expanded zone of suprabasal Cldns was evident - Cldn6 (**B**), Cldn11 (**C**), Cldn12 (**D**) and Cldn18 (**E**). These abnormalities in were strikingly exacerbated in the dermatitis-affected epidermis. For instance, the basal and lower suprabasal layers were void of Cldn1 localization (**F**). In addition, Cldn6 (**G**), Cldn11 (**H**), Cldn12 (**I**) and Cldn18 (**J**) localization was observed throughout the expanded suprabasal zone with membranous localization less evident in the lower suprabasal layers.

Cldn expression was also modified with striking anomalies consistent with those we reported earlier in an acute irritant (12-O-tetradecanoylphorbol-13-acetate (TPA))-induced epidermal injury response [23]. The number of Cldn1-positive basal and suprabasal cells was reduced (**Figure 6A**) and an expanded suprabasal zone of Cldn6- (**Figure 6B**), Cldn11- (**Figure 6C**), Cldn12- (**Figure 6D**) and Cldn18-positive cells (**Figure 6E**) was seen in the transgenic epidermis; concomitantly, a shift away from a strictly membrane localization was evident. Cldn2, Cldn3 and Cldn5 were not expressed in either wild type or transgenic animals (not shown).

**Figure 7:**
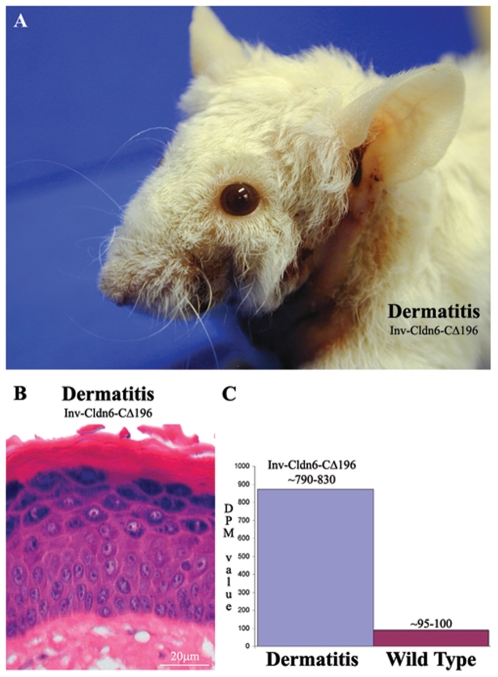
Aging-associated barrier dysfunction. Upon gross observation, obvious signs of dermatitis were evident in aging Inv-Cldn6-CΔ196 transgenic mice, especially in areas subjected to repetitive mechanical stress such as the neck and behind the ears (**A**). Histological evaluation revealed a significantly thickened epidermis with an increased number of spinous/suprabasal cell layers and cellular disorganization; the upper differentiation layers were also abnormal, with parakeratosis, the prevalent appearance of nuclei, an improperly packed granular layer and a thicker stratum corneum (**B**). TEWL measurements across the dermatitis-affected transgenic skin confirmed an approximately 8-fold increase in water loss and a barrier deficient phenotype (**C**).

Taken together, the data suggest an intrinsic propensity for injury and inefficient repair that increases with aging in the Inv-Cldn6-CΔ196 epidermis. This is further supported by two lines of evidence. First, histological and biochemical changes resulting from TPA treatment were seen earlier (4 hours versus 12 hours) and repaired more slowly (no repair versus normalization by 96 hours) in Inv-Cldn6-CΔ196 versus wild type epidermis when mice were tested at 1 month of age (data not shown). Second, obvious signs of dermatitis, especially in areas subjected to repetitive mechanical stress during grooming (e.g. the neck and behind the ears), were seen in the aging Inv-Cldn6-CΔ196 epidermis (**Figure 7A**). Histological evaluation of skin samples from these regions revealed a significantly thickened epidermis with an increased number of spinous/suprabasal cell layers and cellular disorganization; the upper differentiation layers were also abnormal, with parakeratosis, the prevalent appearance of nuclei, an improperly packed granular layer and a thicker stratum corneum (**Figure 7B**). In the dermatitis-affected areas, K14 (**Figure 5H**) and K5 (not shown) were expressed in an expanded zone far into the suprabasal compartment, and K15 expression was only sporadically expressed (**Figure 5I**). K1 was localized throughout the thickened suprabasal zone of the dermatitis-affected Inv-Cldn6-CΔ196 epidermis, and staining appeared punctate, suggesting a keratin filament bundling defect (**Figure 5J**). K6 was seen consistently throughout the basal and suprabasal layers of the dermatitis-affected epidermis (**Figure 5K**); K17-positive basal and suprabasal cells were somewhat more sporadically seen (**Figure 5L**). Expression compartments for involucrin (**Figure 5M**), loricrin (not shown) and filaggrin (**Figure 5N**) were also expanded, with an obvious packing defect reminiscent of the observed histological abnormalities of the stratum corneum. Abnormalities in Cldn1, Cldn6, Cldn11, Cldn12 and Cldn18 localization observed in the aged Inv-Cldn6-CΔ196 transgenic epidermis (see above) were strikingly exacerbated in the dermatitis-affected epidermis. Cldn1 was completely absent in the basal and lower suprabasal layers, but a strictly membranous localization was preserved in the Cldn1-positive upper suprabasal zone (**Figure 6F**). While the distribution of Cldn6 (**Figure 6G**), Cldn11 (**Figure 6H**), Cldn12 (**Figure 6I**) and Cldn18 (**Figure 6J**) corresponded to the expanded suprabasal compartment of the dermatitis-affected Inv-Cldn6-CΔ196 epidermis, immunostaining was less intense and membranous localization less evident in lower suprabasal layers.

The morphological and biochemical abnormalities described predict altered permeability barrier properties and potentially barrier dysfunction in areas of repeated insult of the aged Inv-Cldn6-CΔ196 transgenic epidermis. In keeping with this hypothesis, TEWL measurements across the dermatitis-affected transgenic skin increased ∼8-fold (DPM in the range of 790–830 versus 95–100 of normal adult skin) (**Figure 7C**).

## Discussion

In this study, we used transgenic mouse technology to expand on our structure-function approach to elucidating the role of the cytoplasmic tail of Cldn6 in epidermal development and the formation and maintenance of skin barrier integrity. We demonstrate that Inv-Cldn6-CΔ196 mice display epidermal differentiation abnormalities at birth that result in slower epidermal maturation but are apparently normalized by 1-month of age. However, with aging, intrinsic properties of the Inv-Cldn6-CΔ196 epidermis are manifested by a propensity for injury and inefficient repair, eventually resulting in chronic dermatitis especially in areas subjected to frequent insult via grooming. Changes in Cldn expression and localization suggest marked changes in the presence and function of TJs, as also evidenced by skin barrier dysfunction. Taken together, our data suggest that the overexpression of a Cldn tail truncation mutant in the suprabasal compartment of the mouse epidermis results in its delayed maturation, a propensity for injury, diminished repair and skin barrier deficiency reminiscent of the intrinsic aging process of human skin.

It is well established that the formation of the epidermal permeability barrier (EPB) is developmentally regulated [16], [28]–[30] and that disruption or delay in its formation before birth has a critical role in the survival of the organism [31]–[34]. Previously we reported that a perturbation of Cldn6 expression levels at its endogenous site of expression - the suprabasal layer of the epidermis - alters epidermal development and the formation of the EPB [15], [16]. However, depending on whether normal or mutant forms of Cldn6 are expressed and at what levels, the severity of phenotypic anomalies varies markedly [15]–[17], [19]. For example, perinatal lethality resulting from a severely compromised epidermal differentiation program and dysfunctional EPB results when native Cldn6 is expressed at high levels [16]; while with lower levels of expression, less severe defects occur with the capacity for normalization of EPB function [15]. In contrast, apparently normal prenatal epidermal development and formation of a functioning EPB, but an abnormal postnatal lifelong keratinocyte hyperproliferation leading to progressive thickening of the epidermis - but no dermatitis, results when a mutant form of Cldn6 lacking its entire tail domain (Inv-Cldn6-CΔ187) is expressed [17]. On the other hand, high overexpression of a different mutant lacking only the C-terminal half of the tail domain of Cldn6 (Inv-Cldn6-CΔ196) results in a lethal barrier dysfunction with marked hyperproliferative squamous invaginations/cysts replacing hair follicles [19]. We now show that in Inv-Cldn6-CΔ196 mice with lower levels of transgene expression the abnormalities are much more subtle, and manifested by a slower than usual (i.e., 1 month versus 2 week) but otherwise apparently normal maturation of the epidermis from the multilayered structure seen at birth to the thinner 2–3 cell layer mature structure [15]. These early postnatal changes in maturation are not accompanied by abnormalities in the formation of a functional skin barrier. It is interesting to note that K6- and K17-positive suprabasal cells, which are normally not present in the mature epidermis except in situations of abnormal cell proliferation and differentiation [25]–[27], are not detectable in the interfollicular epidermis of either the juvenile or aged Inv-Cldn6-CΔ196 transgenic samples, until patent dermatitis is evident (see below).

Phenotypic differences observed in the different transgenic mice appear to reflect different mechanistic consequences of expression of native or mutant Cldn6. Structure-function details of different Cldns are only beginning to emerge, but the COOH-terminal tail varies considerably in length and is the region with the most sequence heterogeneity among Cldn isoforms, and in most cases contains a PDZ-binding motif that enables Cldns to directly interact with the TJ-associated MAGUKs (ZO-1, ZO-2, and ZO-3) as well as with MUPP1 and PATJ [35]–[38]. For at least some Cldns (e.g., 1 and 5), the cytoplasmic tail upstream of the PDZ-binding motif is thought to be required for targeting to the TJ complex [39] and contributes to protein stability [17], [40]. Post-translational modifications within the tail domain, including phosphorylation and palmitoylation, are also thought to regulate Cldn activities, including their targeting to the membrane and insertion into TJs [41], but to date most Cldns have not been subjected to exhaustive analysis. Thus, the similarities and differences between Inv-Cldn6-CΔ187 and Inv-Cldn6-CΔ196 mice are of interest. Our data suggest that the phenotype observed in the Inv-Cldn6-CΔ187 epidermis reflects protein instability and an unfolding protein response. On the other hand, the delayed maturation and age-related deficit in wound healing and repair in the Inv-Cldn6-CΔ196 epidermis most likely involves an aberrant interaction with the cytoskeleton. Although to date no direct interaction between the cytoplasmic tail of Cldns and cytoskeletal molecules has been shown, the possibility of such direct interactions is not unlikely considering the known role of the actin cytoskeleton, for example, in cell shape and cell polarity [42]–[44].

Despite the importance of the skin barrier to the survival of the mammalian species, the molecular mechanisms governing its formation and maintenance are not well-understood, nor are the changes that occur during the intrinsic aging process of the skin. In humans, aging-related changes in the skin are widespread and result from both intrinsic (gene mutations, hormonal changes, cellular metabolism) and extrinsic (UV exposure, chemicals, pollutants) factors. While extrinsic factors affect mainly the dermis, the epidermis is the main target of intrinsic skin aging processes where, especially in sun-exposed skin, changes occur often with initial generalized epidermal thickening, indicative of epidermal hyperplasia and reduced capacity to repair, such that the epidermis becomes prone to a number of skin conditions including dermatitis, eczema and ulceration [6], [7], [45]–[49]. Only recently has it been appreciated that abnormal skin barrier function might underlie these skin conditions [50], although no analysis of Cldn expression has been reported. The murine epidermis, however, does not normally undergo comparable aging-related changes; in fact, once the mouse epidermis is matured it maintains a homeostatic balance and undergoes morphological and biochemical changes only in response to injury or disease. Thus, it was extremely interesting to note that in spite of the fact that Inv-Cldn6-CΔ196 mice achieve a normal-appearing epidermis by one-month of age (by morphology and biochemical markers), with aging they have a high propensity for epidermal injury and a diminished ability to repair lesions, leading eventually to severe dermatitis with associated changes in skin barrier function. It should be noted that this sensitivity to injury and inefficient repair were seen also in young mice subjected to acute injury by application of the irritant TPA (data not shown).

As already indicated, the precise mechanisms by which expression of low levels of Cldn6-CΔ196 in the suprabasal layers of the epidermis leads to age-related changes appearing to mimic those seen in humans are currently not known, but our observations suggest that changes in Cldn homeostasis leading to changes in the epidermal differentiation program play important roles. In this regard, not only changes elicited directly by changes in Cldn6 signaling but also changes imparted by alterations in the expression of other Cldns, notably Cldn1, must be considered. In the developing epidermis, Cldn1 is first restricted to the stratifying layers and matures to occupy the basal layer upon the completion of barrier formation at E17.5 [19]. However, in response to TPA-induced injury and loss of cell polarity seen in tumorigenesis, Cldn1 expression is downregulated in both the basal layer and immediate suprabasal layers of the epidermis [23], [51], alterations seen also with aging of the Inv-Cldn6-CΔ196 epidermis. The drastic changes in TEWL that we observed in severely affected areas of the Inv-Cldn6-CΔ196 epidermis also appear to mimic those seen in the aging human epidermis subjected to stress or injury [47]. Thus the data reported here suggest that the changes in Cldn expression and localization in mice with low expression of Cldn6-CΔ196 in the epidermis lead to relatively subtle changes in the epidermal differentiation program and permeability in the young animal, but render them prone to injury and diminished repair that are exacerbated especially in areas subjected to repeated mechanical trauma, leading to chronic and increasingly severe dermatitis.

In summary, our data add to the evidence that Cldn6, and in particular the C-terminal half of the Cldn6 tail, contribute to the regulation of epidermal differentiation and skin barrier function throughout life. They also support the utility of the Inv-Cldn6-CΔ196 mouse model for studies aimed at understanding intrinsic changes in the aging epidermis and point towards a need to investigate Cldn expression profiles in the aging human epidermis.

## References

[1] Fuchs E, Raghavan S (2002). Getting under the skin of epidermal morphogenesis.. Nat Rev Genet.

[2] Fuchs E (2007). Scratching the surface of skin development.. Nature.

[3] Turksen K, Troy TC (1998). Epidermal cell lineage.. Biochem Cell Biol.

[4] Pouillot A, Dayan N, Polla AS, Polla LL, Polla BS (2008). The stratum corneum: a double paradox.. J Cosmet Dermatol.

[5] Elias PM, Williams ML, Holleran WM, Jiang YJ, Schmuth M (2008). Pathogenesis of permeability barrier abnormalities in the ichthyoses: inherited disorders of lipid metabolism.. J Lipid Res.

[6] Elias PM, Ghadially R (2002). The aged epidermal permeability barrier: basis for functional abnormalities.. Clin Geriatr Med.

[7] Ghadially R (1998). Aging and the epidermal permeability barrier: implications for contact dermatitis.. Am J Contact Dermat.

[8] Farquhar MGaPGE (1963). Junctional complexes in various epithelia.. J Cell Biol.

[9] Krause G, Winkler L, Mueller SL, Haseloff RF, Piontek J (2008). Structure and function of claudins.. Biochim Biophys Acta.

[10] Paris L, Tonutti L, Vannini C, Bazzoni G (2008). Structural organization of the tight junctions.. Biochim Biophys Acta.

[11] Chiba H, Osanai M, Murata M, Kojima T, Sawada N (2008). Transmembrane proteins of tight junctions.. Biochim Biophys Acta.

[12] Turksen K, Troy TC (2004). Barriers built on claudins.. J Cell Sci.

[13] Katoh M (2003). CLDN23 gene, frequently down-regulated in intestinal-type gastric cancer, is a novel member of CLAUDIN gene family.. Int J Mol Med.

[14] Furuse M, Hata M, Furuse K, Yoshida Y, Haratake A (2002). Claudin-based tight junctions are crucial for the mammalian epidermal barrier: a lesson from claudin-1-deficient mice.. J Cell Biol.

[15] Troy TC, Rahbar R, Arabzadeh A, Cheung RM, Turksen K (2005). Delayed epidermal permeability barrier formation and hair follicle aberrations in Inv-Cldn6 mice.. Mech Dev.

[16] Turksen K, Troy TC (2002). Permeability barrier dysfunction in transgenic mice overexpressing claudin 6.. Development.

[17] Arabzadeh A, Troy TC, Turksen K (2006). Role of the cldn6 cytoplasmic tail domain in membrane targeting and epidermal differentiation in vivo.. Mol Cell Biol.

[18] Gonzalez-Mariscal L, Betanzos A, Nava P, Jaramillo BE (2003). Tight junction proteins.. Prog Biophys Mol Biol.

[19] Troy TC, Turksen K (2007). The targeted overexpression of a Claudin mutant in the epidermis of transgenic mice elicits striking epidermal and hair follicle abnormalities.. Mol Biotechnol.

[20] Troy TC, Li Y, O'Malley L, Turksen K (2007). The temporal and spatial expression of Claudins in epidermal development and the accelerated program of epidermal differentiation in K14-CaSR transgenic mice.. Gene Expr Patterns.

[21] Hohl D, Mehrel T, Lichti U, Turner ML, Roop DR (1991). Characterization of human loricrin. Structure and function of a new class of epidermal cell envelope proteins.. J Biol Chem.

[22] Troy TC, Turksen K (2005). Commitment of embryonic stem cells to an epidermal cell fate and differentiation in vitro.. Dev Dyn.

[23] Arabzadeh A, Troy TC, Turksen K (2008). Claudin expression modulations reflect an injury response in the murine epidermis.. J Invest Dermatol.

[24] Fuchs E, Byrne C (1994). The epidermis: rising to the surface.. Curr Opin Genet Dev.

[25] Leigh IM, Navsaria H, Purkis PE, McKay IA, Bowden PE (1995). Keratins (K16 and K17) as markers of keratinocyte hyperproliferation in psoriasis in vivo and in vitro.. Br J Dermatol.

[26] McGowan K, Coulombe PA (1998). The wound repair-associated keratins 6, 16, and 17. Insights into the role of intermediate filaments in specifying keratinocyte cytoarchitecture.. Subcell Biochem.

[27] McGowan KM, Coulombe PA (1998). Onset of keratin 17 expression coincides with the definition of major epithelial lineages during skin development.. J Cell Biol.

[28] Hardman MJ, Sisi P, Banbury DN, Byrne C (1998). Patterned acquisition of skin barrier function during development.. Development.

[29] Byrne C, Hardman MJ (2005). Whole-mount assays for gene induction and barrier formation in the developing epidermis.. Methods Mol Biol.

[30] Hardman MJ, Moore L, Ferguson MW, Byrne C (1999). Barrier formation in the human fetus is patterned.. J Invest Dermatol.

[31] Elias PM (2005). Stratum corneum defensive functions: an integrated view.. J Invest Dermatol.

[32] Mack JA, Anand S, Maytin EV (2005). Proliferation and cornification during development of the mammalian epidermis.. Birth Defects Res C Embryo Today.

[33] Williams ML, Hanley K, Elias PM, Feingold KR (1998). Ontogeny of the epidermal permeability barrier.. J Investig Dermatol Symp Proc.

[34] Cartlidge P (2000). The epidermal barrier.. Semin Neonatol.

[35] Assemat E, Bazellieres E, Pallesi-Pocachard E, Le Bivic A, Massey-Harroche D (2008). Polarity complex proteins.. Biochim Biophys Acta.

[36] Roh MH, Margolis B (2003). Composition and function of PDZ protein complexes during cell polarization.. Am J Physiol Renal Physiol.

[37] Heiskala M, Peterson PA, Yang Y (2001). The roles of claudin superfamily proteins in paracellular transport.. Traffic.

[38] Koval M (2006). Claudins–key pieces in the tight junction puzzle.. Cell Commun Adhes.

[39] Ruffer C, Gerke V (2004). The C-terminal cytoplasmic tail of claudins 1 and 5 but not its PDZ-binding motif is required for apical localization at epithelial and endothelial tight junctions.. Eur J Cell Biol.

[40] Van Itallie CM, Gambling TM, Carson JL, Anderson JM (2005). Palmitoylation of claudins is required for efficient tight-junction localization.. J Cell Sci.

[41] Simard A, Di Pietro E, Young CR, Plaza S, Ryan AK (2006). Alterations in heart looping induced by overexpression of the tight junction protein Claudin-1 are dependent on its C-terminal cytoplasmic tail.. Mech Dev.

[42] Ivanov AI (2008). Actin motors that drive formation and disassembly of epithelial apical junctions.. Front Biosci.

[43] Ivanov AI, McCall IC, Parkos CA, Nusrat A (2004). Role for actin filament turnover and a myosin II motor in cytoskeleton-driven disassembly of the epithelial apical junctional complex.. Mol Biol Cell.

[44] Miyoshi J, Takai Y (2008). Structural and functional associations of apical junctions with cytoskeleton.. Biochim Biophys Acta.

[45] Makrantonaki E, Zouboulis CC (2007). Molecular mechanisms of skin aging: state of the art.. Ann N Y Acad Sci.

[46] Ward S (2005). Eczema and dry skin in older people: identification and management.. Br J Community Nurs.

[47] Ghadially R, Brown BE, Sequeira-Martin SM, Feingold KR, Elias PM (1995). The aged epidermal permeability barrier. Structural, functional, and lipid biochemical abnormalities in humans and a senescent murine model.. J Clin Invest.

[48] Davies A (2008). Management of dry skin conditions in older people.. Br J Community Nurs.

[49] Kligman LH (1989). Photoaging. Manifestations, prevention, and treatment.. Clin Geriatr Med.

[50] Barland CO, Zettersten E, Brown BS, Ye J, Elias PM (2004). Imiquimod-induced interleukin-1 alpha stimulation improves barrier homeostasis in aged murine epidermis.. J Invest Dermatol.

[51] Arabzadeh A, Troy TC, Turksen K (2007). Changes in the distribution pattern of Claudin tight junction proteins during the progression of mouse skin tumorigenesis.. BMC Cancer.

